# Verification of hub genes in the expression profile of aortic dissection

**DOI:** 10.1371/journal.pone.0224922

**Published:** 2019-11-21

**Authors:** Weitie Wang, Qing Liu, Yong Wang, Hulin Piao, Bo Li, Zhicheng Zhu, Dan Li, Tiance Wang, Rihao Xu, Kexiang Liu

**Affiliations:** 1 Department of Cardiovascular Surgery, Second Hospital of Jilin University, Changchun, Jilin, China; 2 Graduate School of Medicine and Faculty of Medicine, University of Tokyo, Tokyo, Japan; Albany Medical College, UNITED STATES

## Abstract

**Background:**

To assess the mRNA expression profile and explore the hub mRNAs and potential molecular mechanisms in the pathogenesis of human thoracic aortic dissection (TAD).

**Methodology:**

mRNA microarray expression signatures of TAD tissues (n = 6) and non-TAD tissues (NT; n = 6) were analyzed by an Arraystar human mRNA microarray. Real-time PCR (qRT-PCR) was used to validate the results of the mRNA microarray. Bioinformatic tools, including Gene Ontology and Kyoto Encyclopedia of Genes and Genomes pathway analysis, were utilized. Protein-protein interaction (PPI) networks were constructed based on data from the STRING database. Molecular Complex Detection (MCODE) and cytoHubba analyses were used to predict the strongest hub gene and pathway.

**Results:**

The top 10 hub genes were CDK1, CDC20, CCNB2, CCNB1, MAD2L1, AURKA, C3AR1, NCAPG, CXCL12 and ASPM, which were identified from the PPI network. Module analysis revealed that TAD was associated with the cell cycle, oocyte meiosis, the p53 signaling pathway, and progesterone-mediated oocyte maturation. The qRT-PCR results showed that the expression of all hub genes was significantly increased in TAD samples (p < 0.05). Immunostaining of Ki-67 and CDK1 showed a high proliferation state and high expression in TAD, respectively.

**Conclusions:**

CDK1 could be used as a potential diagnostic biomarker and therapeutic target of TAD.

## Introduction

Thoracic aortic dissection (TAD) is a common and life-threatening aortic disease [1]. Despite improvements in medical therapy and surgical or endovascular techniques in recent years, TAD still has a high morbidity and mortality rate [2]. Owing to the poor results of existing treatment methods, further understanding of the molecular mechanism may provide new insights into therapeutic targets for TAD. Many reports have shown that the degradation of extracellular matrix (ECM) and depletion of vascular smooth muscle cells (VSMCs) of the aortic wall are the main histopathological findings [3–5]. However, the key molecular mechanism of TAD pathogenesis remains unclear.

In recent years, mRNAs have been reported to participate in the regulation of pathophysiological conditions and have been shown to be involved in the progression of cardiovascular disease [6]. Currently, research has focused on genome-wide association studies (GWAS) [7], which could identify relevant genetic variants that may be used as potential biomarkers for diagnosis and targeted therapy. Although high-throughput sequencing technology has identified many genes with diverse expression, different expression profiles in TAD have provided various results, and no reliable results have been identified until now [8–10]. More samples and advanced bioinformatics methods should be used in further analyses.

In the present study, we used mRNA microarrays to acquire differential expression profiles in human TAD tissues and coronary heart disease (CAD) tissues. Subsequently, biomathematical analysis was used to explore the potential functions, identify the hub genes and explore the intrinsic molecular mechanisms involved in TAD. Quantitative reverse-transcription PCR (qRT-PCR) and immunostaining of cyclin-dependent kinase 1 (CDK1) showed high expression in TAD, providing a foundation for new biomarker and therapeutic targets for human TAD.

## Results

### Differential expression profiles of mRNAs in the TAD and NT Groups

**Fig 1:**
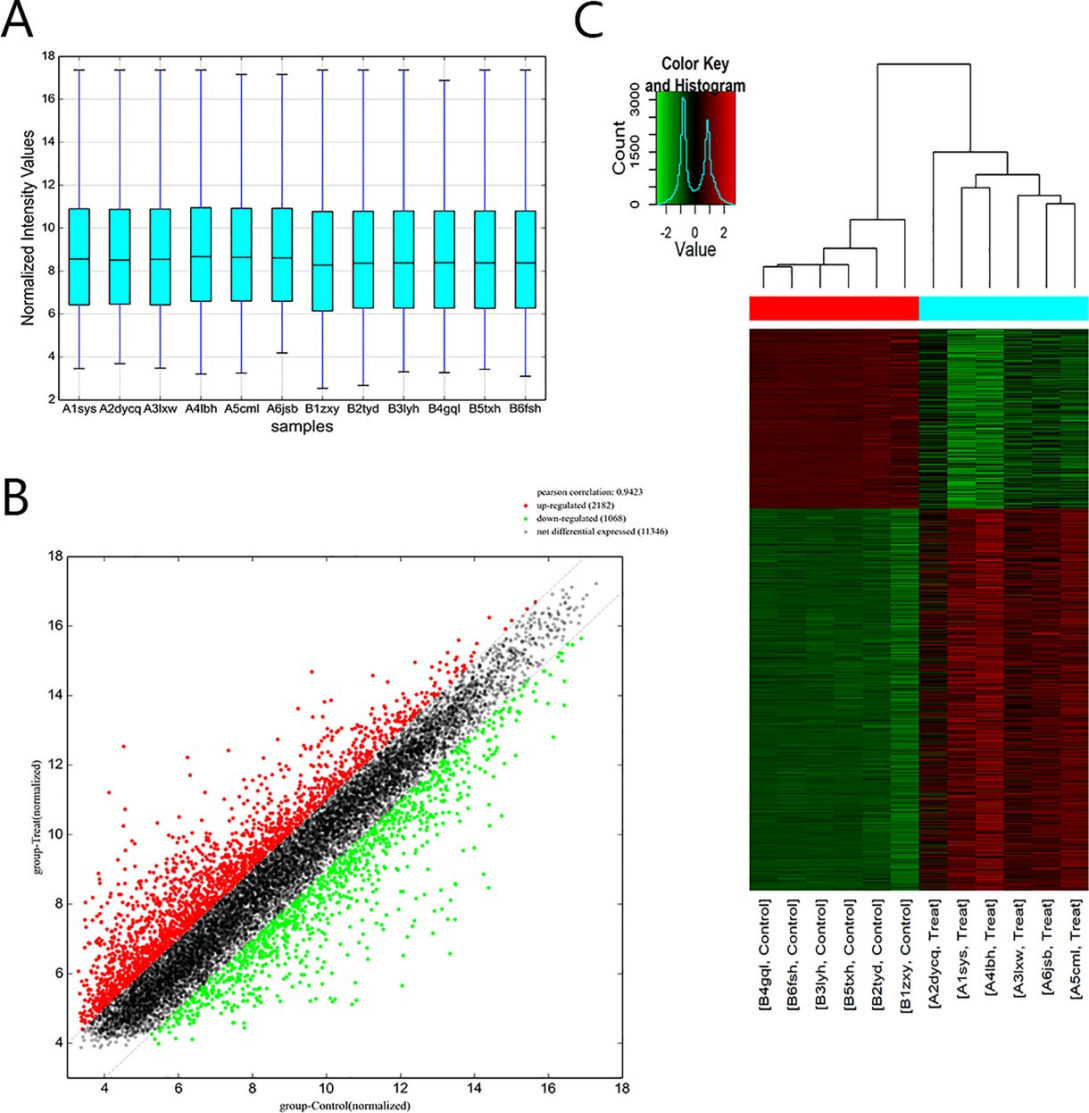
Comparison of mRNA expression profiles between the TAD samples and NT samples. Comparison of mRNA expression profiles between the TAD samples and NT samples. (A) The box plot is a convenient method to quickly compare the distribution of mRNAs. After normalization, the distributions of log2 ratios among the tested samples were almost similar. (B) The scatterplot is a visualization method that is useful for assessing the variation between TAD and control tissues compared by microarrays. The values of the X and Y axes in the scatterplot are averaged normalized values in each group (log 2 scaled). The green plot shows a decrease, and the red plot shows an increase in different genes. (C) Differentially expressed genes can be effectively divided into the TAD and NT groups. Red indicates that the gene that is upregulated, and green represents downregulated genes.

Volcano plots revealed that mRNAs were differentially expressed in human TAD aortic tissues through microarray technology (Fig 1). A total of 2834 mRNAs were differentially expressed in the TAD group relative to the NT group. In total, 1928 mRNAs were upregulated, and 906 mRNAs were downregulated (fold change >2.0, P-value < 0.05). The top ten up- and downregulated mRNAs are listed in Table 1.

**Table 1:** The top ten up- and downregulated mRNAs.

Upregulated			Downregulated		
Gene Symbol	FDR	P-value	Gene Symbol	FDR	P-value
MARCO	258.8940442	9.67102E-06	ATXN3L	108.3473569	2.30333E-05
CHI3L2	135.8107372	0.00011992	SLC51B	83.5617528	2.52205E-06
CCL18	72.0452345	3.67575E-05	GYS1	60.3188012	7.75796E-06
SPP1	62.9254504	0.000147395	KITLG	60.045535	6.2348E-06
SAA2	53.30226	2.78619E-05	C8orf12	52.4563995	7.96212E-05
APOC1	42.1502422	7.24249E-05	OR6A2	52.3701259	3.53724E-05
SERPINA3	33.7417914	2.36918E-07	AC093323.1	47.6062725	2.71921E-06
MT1G	33.6791872	0.000460576	ITLN1	47.4071743	1.01876E-07
CHRDL2	33.6103171	1.26255E-05	ZNF367	46.8660148	6.64981E-06
RRM2	32.9976749	0.000779975	OFCC1	41.6471558	6.39121E-05

### GO functional enrichment analysis

**Fig 2:**
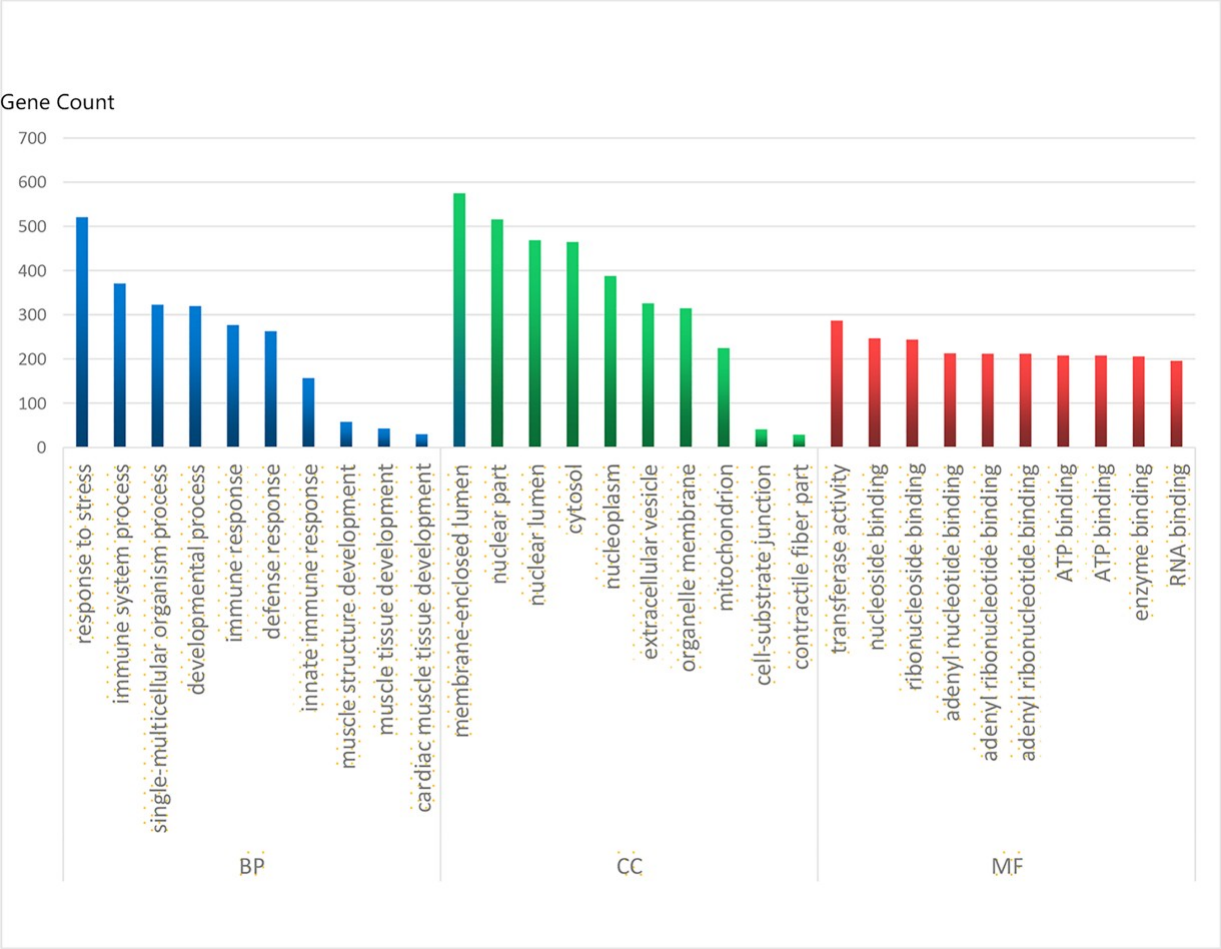
Gene Ontology analysis classified the differentially expressed genes into 3 groups. Molecular function, biological process, and cellular component.

To determine the functions of all DEGs, we used the Database for Annotation, Visualization, and Integrated Discovery (DAVID) online tool, and the DEG functions of GO function enrichment were divided into three groups: biological processes (BP), cellular component (CC), and molecular function (MF) (Fig 2). As shown in Fig 2 and Table 2, in the BP group, the downregulated DEGs were mainly enriched in cardiac muscle tissue development, muscle tissue development, muscle structure development, developmental process, and single-multicellular organism process, and the upregulated DEGs were mainly enriched in response to stress, defense response, immune system process and innate immune response. In the CC group, the downregulated DEGs were mainly enriched in contractile fiber, myofibril, sarcomere, contractile fiber part and cell-substrate junction, and the upregulated DEGs were mainly enriched in cytoplasm, cytoplasmic part, intracellular organelle part, organelle part and membrane-bounded organelle. In the MF group, the downregulated DEGs were mainly enriched in protein phosphatase regulator activity, phosphatase regulator activity, cytoskeletal protein binding, structural constituents of muscle and actin binding, and the upregulated DEGs were mainly enriched in catalytic activity, protein binding, single-stranded DNA-dependent ATPase activity, ATP binding and adenyl ribonucleotide binding.

**Table 2:** The significantly enriched analysis of differentially expressed genes in thoracic aortic dissection.

Expression	Category	Term	Description	Gene Count	P-Value
DOWN-DEGs	BP	cardiac muscle tissue development	GO:0048738	30	1.43E-10
	BP	muscle tissue development	GO:0060537	43	2.41E-10
	BP	muscle structure development	GO:0061061	58	4.80E-10
	BP	developmental process	GO:0032502	320	1.23E-09
	BP	single-multicellular organism process	GO:0044707	323	2.13E-09
	CC	contractile fiber	GO:0043292	32	6.27E-10
	CC	myofibril	GO:0030016	30	2.86E-09
	CC	sarcomere	GO:0030017	28	3.41E-09
	CC	contractile fiber part	GO:0044449	29	5.76E-09
	CC	cell-substrate junction	GO:0030055	41	1.51E-07
	MF	protein phosphatase regulator activity	GO:0019888	13	1.20E-05
	MF	phosphatase regulator activity	GO:0019208	14	1.39E-05
	MF	cytoskeletal protein binding	GO:0008092	57	2.60E-04
	MF	structural constituent of muscle	GO:0008307	8	2.74E-04
	MF	actin binding	GO:0003779	32	2.77E-04
UP-DEGs	BP	immune response	GO:0006955	277	4.29E-26
	BP	response to stress	GO:0006950	521	1.53E-24
	BP	defense response	GO:0006952	263	1.99E-23
	BP	immune system process	GO:0002376	371	2.30E-22
	BP	innate immune response	GO:0045087	157	5.71E-17
	CC	cytoplasm	GO:0005737	1233	7.68E-33
	CC	cytoplasmic part	GO:0044444	983	4.18E-31
	CC	intracellular organelle part	GO:0044446	984	5.62E-28
	CC	organelle part	GO:0044422	995	1.89E-26
	CC	membrane-bounded organelle	GO:0043227	1323	6.40E-24
	MF	catalytic activity	GO:0003824	695	1.76E-12
	MF	protein binding	GO:0005515	1006	3.26E-11
	MF	single-stranded DNA-dependent ATPase activity	GO:0043142	10	4.99E-09
	MF	ATP binding	GO:0005524	208	2.82E-08
	MF	adenyl ribonucleotide binding	GO:0032559	212	3.10E-08

### Signaling pathway analysis

**Fig 3:**
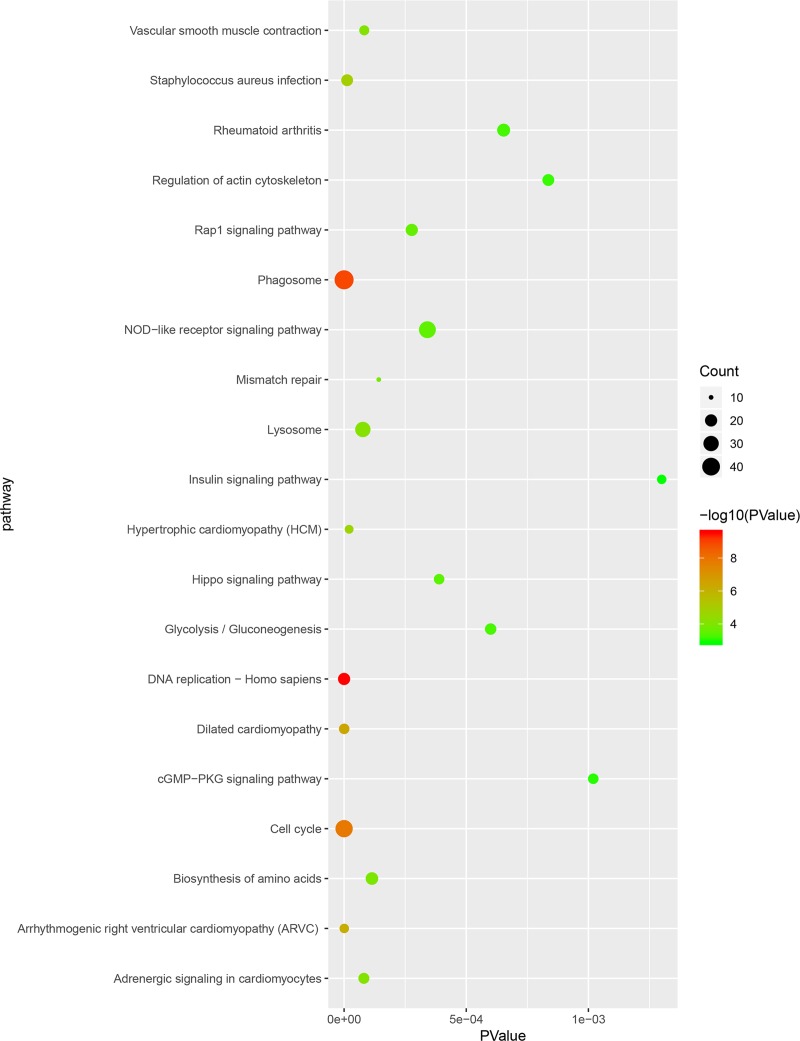
Kyoto Encyclopedia of Genes and Genomes enrichment analysis of the pathways. The gradient color represents the P-value; the size of the black spots represents the gene number.

**Fig 4:**
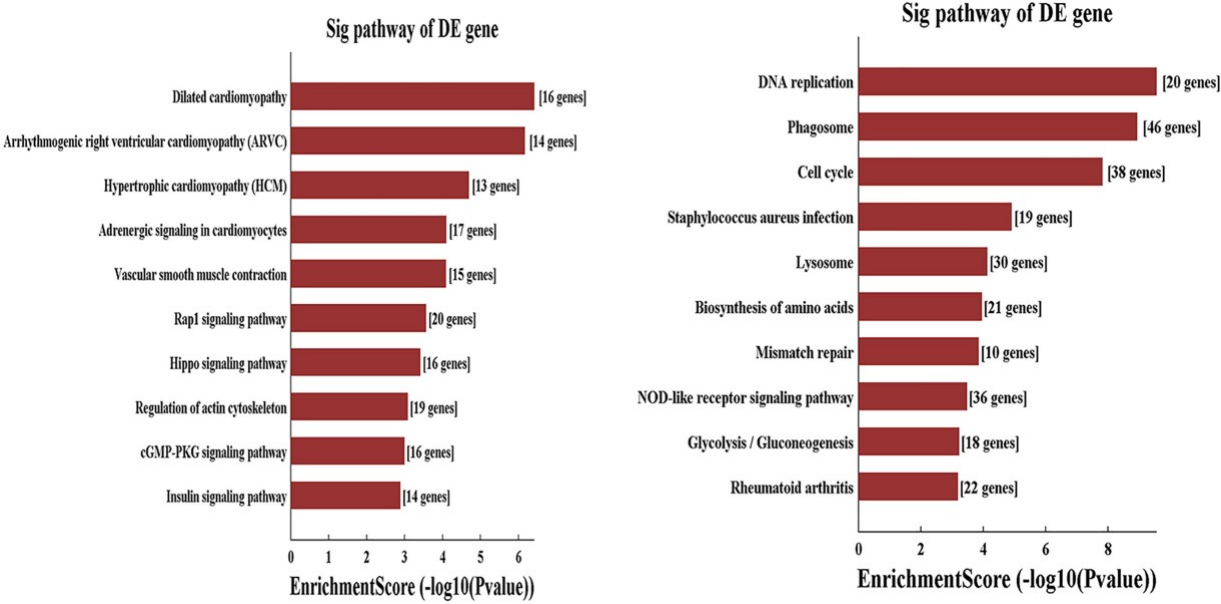
Significantly upregulated and downregulated pathways.

After the pathway enrichment analysis, the downregulated genes were mainly enriched in dilated cardiomyopathy, arrhythmogenic right ventricular cardiomyopathy, hypertrophic cardiomyopathy, adrenergic signaling in cardiomyocytes and vascular smooth muscle contraction. The upregulated genes were mainly enriched in DNA replication, phagosome, cell cycle, Staphylococcus aureus infection and lysosome (Figs 3 and 4).

### Protein-Protein Interaction (PPI) network and modular analysis

**Fig 5:**
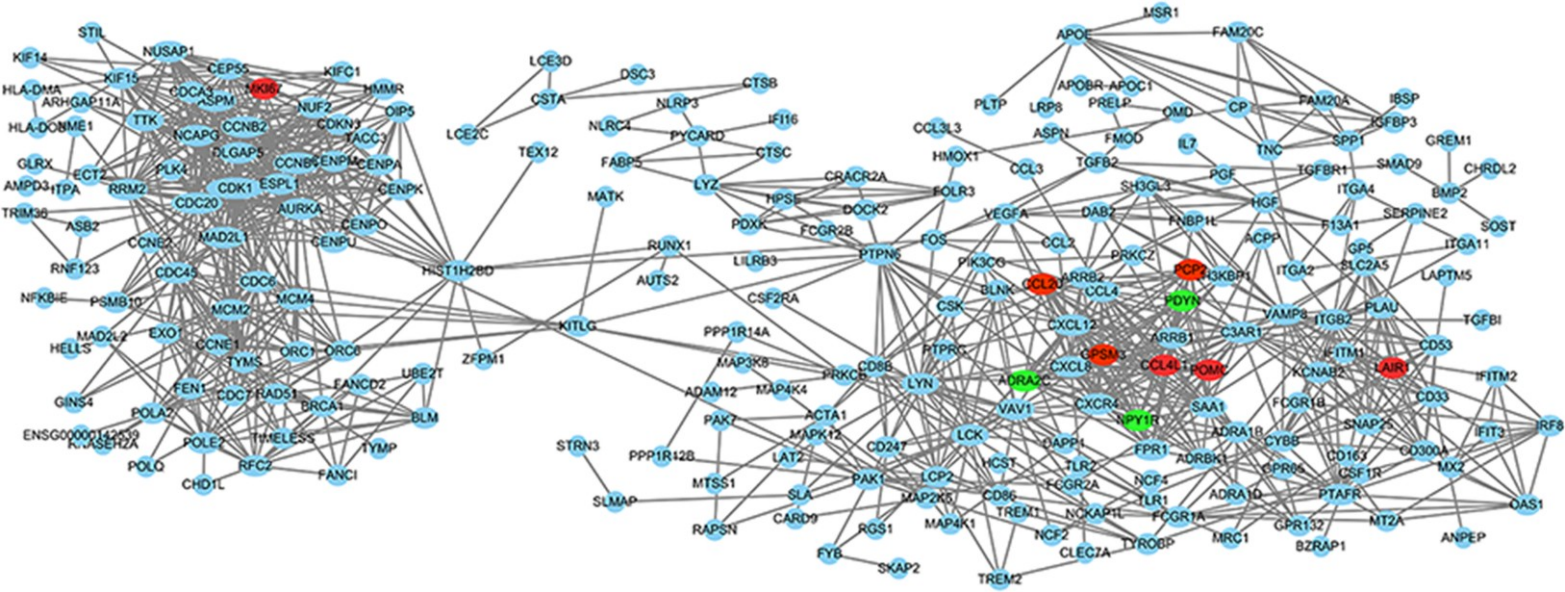
PPI network constructed with the differentially expressed genes. PPI network constructed with the differentially expressed genes. Red nodes represent hub GENE analysis by cytoHubba.

**Fig 6:**
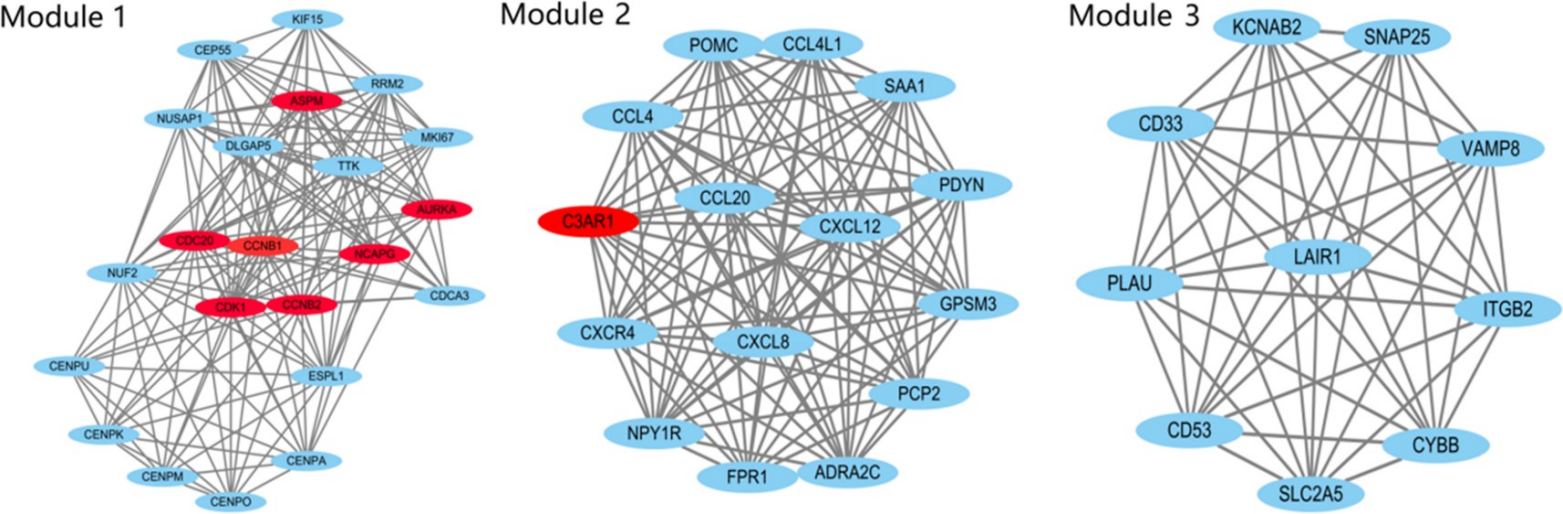
The three most significant modules. Red nodes represent hub GENE analysis by cytoHubba.

All the DEGs (fold change >4.0) were analyzed using the STRING database. Then, we analyzed these data by using Cytoscape software to construct a PPI network containing 433 nodes and 348 edges (Fig 5). In these DEGs, 10 hub genes, including CDK1, CDC20, CCNB2, CCNB1, MAD2L1, AURKA, C3AR1, NCAPG, CXCL12, and ASPM, were identified after calculation. Among these 10 hub genes, CDK1 presented the highest degree (degree = 44). The Cytoscape plugin MCODE showed the top three modules with scores of 15.429, 14.857 and 10.000 (Fig 6). Then, the genes in these three modules were analyzed by functional enrichment. Pathway enrichment analysis showed that Module 1 was mainly relevant to cell cycle oocyte meiosis, the p53 signaling pathway, and progesterone-mediated oocyte maturation. Module 2 was mainly associated with the chemokine signaling pathway, cytokine-cytokine receptor interaction, rheumatoid arthritis, NF-kappa B signaling pathway, and neuroactive ligand-receptor interaction. Module 3 was mainly associated with leukocyte transendothelial migration.

### Validation by qRT-PCR of differentially expressed mRNAs

**Fig 7:**
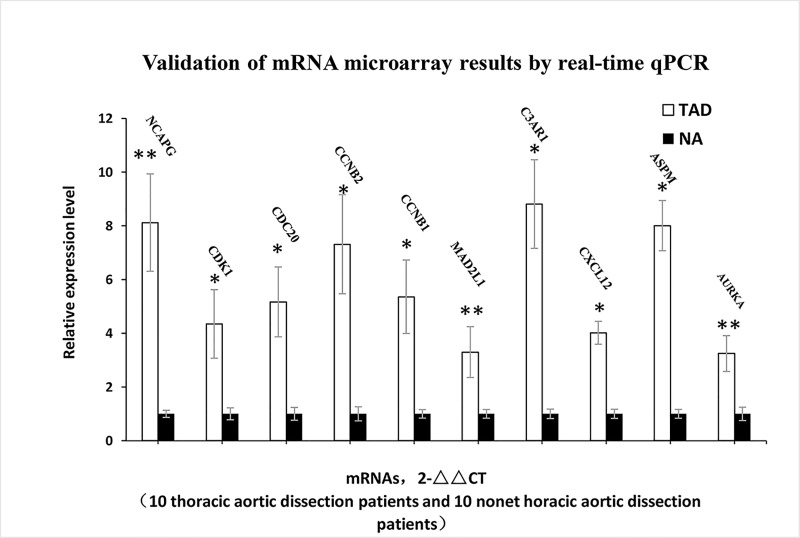
Validation of the mRNA microarray results by real-time qPCR. mRNA microarray results were verified by real-time qPCR between the TAD group (n = 10) and the NT group (n = 10). All samples were normalized to the expression of GAPDH, and the relative expression levels of each gene were analyzed using the 2-ΔΔCt method. *P < 0.05, **P < 0.01.

To validate the microarray results, the expression levels of the top 10 hub genes were determined in ascending aortic samples of thoracic aortic dissection and no thoracic aortic dissection using qRT-PCR. The verification results showed that the expression levels of the 10 hub genes were significantly increased in thoracic aortic dissection samples (p < 0.05) (Fig 7). All validation data were consistent with the microarray data and analytical results in this study.

### Immunostaining of Ki-67 and CDK1

**Fig 8:**
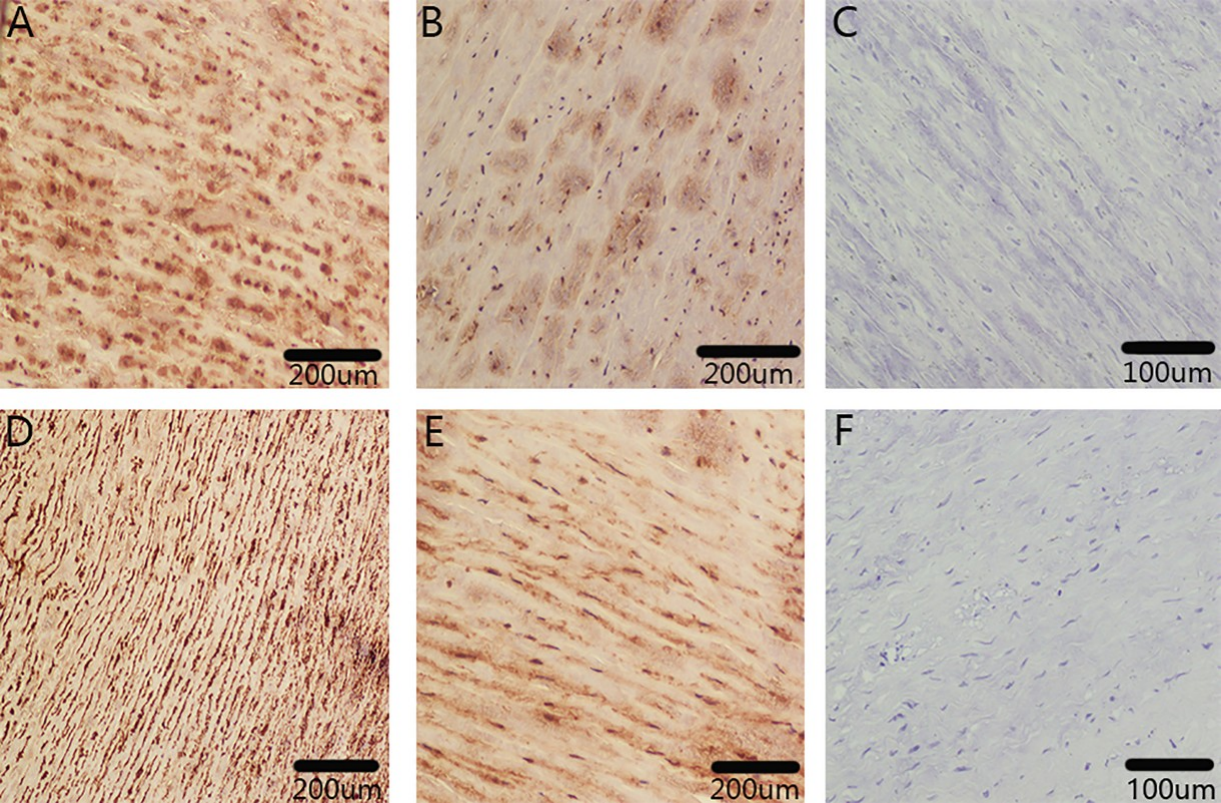
Immunostaining of Ki-67 and CDK1. Immunohistochemical analysis of Ki-67 and CDK1 expression in aortic dissection (n = 5) and control tissue samples (n = 5). A: Strong Ki-67 expression on aortic dissection tissue. B: Weak Ki-67 expression on control tissue. C: Secondary-antibody-only negative control. D: Strong CDK1 expression on aortic dissection tissue. E: Weak CDK1 expression on control tissue. F: Secondary-antibody-only negative control.

Immunostaining identified the strong Ki-67 and CDK1 expression on aortic dissection tissues (Fig 8A and 8D) and weak Ki-67 and CDK1 expression on control tissues (Fig 8B and 8E).

## Discussion

TAD is a life-threatening event that carries a high mortality rate [1]. Patients generally complain of acute chest and back pain and are often misdiagnosed with acute myocardial infarction [11]. Surgical treatment seems to be the best method for this disease [12]. However, the traditional surgical treatment for TAD is very complex and time-consuming with high mortality and poor prognosis. Therefore, it is necessary to research new biomarkers and therapeutic targets. The molecular mechanism of this severe disease remains unclear. TAD, similar to Marfan syndrome and Ehlers-Danlos syndrome, has been confirmed to involve glycoprotein deficiency [13] and shows abnormal type-III precollagen [14], respectively. However, most patients with TAD do not exhibit such explicit syndromes. These TAD patients generally show hypertension, atherosclerosis and trauma [11]. In addition, many studies have indicated that degradation of ECM and depletion of VSMC play important roles in nonhereditary TAD [15]. Therefore, studies are primarily focused on the protein-coding genes of the ECM [16] and VSMCs, such as COL3A1, FBN1, LOX, FLNA, ACTA2, and MYH11. These protein-coding genes play a role in the pathological processes of TAD. However, the key pathogenesis of TAD has not been confirmed until now.

Modern molecular biology has shown that disease is induced by changes in the gene expression profiles of tissues or cells. Collection, summary and analysis of these genes can help us to understand the mechanisms of various diseases. High-throughput sequencing technology has been used to detect the identity of altered genes in tissue and has been widely used to predict potential target genes. To date, many studies have been performed, and thousands of differentially expressed genes (DEGs) have been screened. However, different gene profiles vary with each study [8–10]. Therefore, further analysis should be carried out, and hub genes and pathways should be identified.

This study identified 2834 mRNA DEGs, which included 1928 upregulated DEGs and 906 downregulated DEGs (fold change >2.0, P-value < 0.05). Similar to other studies [8–10], we classified the DEGS into three groups, BP, CC, and MF, by GO terms. GO functional enrichment analysis showed that the top terms were immune response, response to stress, defense response and immune system process, which was consistent with other studies. Inflammatory and immunological factors may both lead to vascular damage to the aortic wall [17–19]. Reports have shown that inflammatory mechanisms participate in medial degeneration of aortic dissection tissue. Macrophages and activated T lymphocytes were also found in the dissection tissue. The T helper 2 response was reported to be relevant to the growth of aneurysms [20–21], and hyperexpression of IL-6 and IL-8 in aortic dissection was also present, indicating that immunologic pathways were critical in aortic wall damage [22]. In addition, neutrophils, CD8+, CD28–, IL-6, TNF- α, IL-8, and MCP-1 suggested that cytotoxic and innate cells were mainly relevant to the pathogenesis of TAD [23]. T helper lymphocytes and activated macrophages, mast cells, T and B lymphocytes in the immune system process and inflammatory pathways were involved in the weakening of the aortic wall.

Furthermore, the enriched Kyoto Encyclopedia of Genes and Genomes (KEGG) pathways of downregulated genes were mainly enriched in dilated cardiomyopathy, arrhythmogenic right ventricular cardiomyopathy, hypertrophic cardiomyopathy, adrenergic signaling in cardiomyocytes and vascular smooth muscle contraction. VSMCs were the major cells in the aortic media. The functions of VSMCs, such as proliferation and migration, are critical for the biomechanical properties of the aortic wall. These parameters were affected by many factors, including lymphocytes that induce the apoptosis of VSMCs and synthesis of MMPs. Therefore, many studies aimed to research the function of VSMCs. Talin-1 has been reported to mainly exist in the aortic media, and it was significantly downregulated in AD aortic tissue. Further analysis confirmed that Talin-1 regulates VSMC proliferation and migration and finally causes pathologic vascular remodeling to alter vascular media structure and function and lead to AD [24]. Many genes, such as YAP1, Sirtuin-1, PCSK9, polycystin-1, and brahma-related gene 1, have also been reported to be associated with the pathophysiologic processes of aortic dissection by influencing the proliferation and migration of VSMCs [25–27].

Similar to previous high-throughput sequencing studies, we also identified many relevant pathways through analysis such as “Dilated cardiomyopathy”, “Arrhythmogenic right ventricular cardiomyopathy” and “Hypertrophic cardiomyopathy,” but we could not determine which mechanism was the most relevant to TAD and the strongest hub gene in AD. The tissue type of TAD most likely does not contain cardiomyocytes. Therefore, we carried out further analyses, such as PPI Network and Modular Analysis, including the STRING database, cytoHubba analysis and MCODE analysis, which are widely used to infer the top hub genes and pathways [28]. In our study, we picked a gene that was differentially expressed up to 4-fold to reduce the scope of the study. The cytoHubba analysis showed that the top ten hub genes were CDK1, CDC20, CCNB2, CCNB1, MAD2L1, AURKA, C3AR1, NCAPG, CXCL12, and ASPM. These genes indicated that the cell cycle played an important role in TAD. Therefore, the Cytoscape plugin MCODE was used to perform analysis from another perspective. After calculation, the top module included 22 nodes and 162 edges. We chose the 22 nodes for functional enrichment analyses. The cell cycle was the most relevant pathway and included 7 hub genes that had been previously predicted. Therefore, we aimed to analyze the hub gene CDK1 and the cell cycle in TAD.

CDK 1 is the founding member of the CDK family in human cells [29]. This molecule is conserved across all leukocytes and is the only essential cell cycle CDK in human cells. One study reported that this important member of the CDK family is required for successful completion of M-phase. In addition, the conserved nature and remodeling function indicate the importance of this molecule. In the cell cycle, CDK1 always acts in combination with cyclin A and cyclin B. Therefore, CDK1 can regulate the cell cycle from GI to S phase because its partner cyclin A is first expressed during late G1, when it initially binds to CDK2 and promotes S-phase [30]. As the CDK family in association with partner cyclin proteins mediates the progression of the cell cycle, CDK1 inhibition leads to the initiation of adhesion remodeling in preparation for entry into mitosis, revealing an intimate link between the cell cycle machinery and cell-ECM adhesion. Therefore, the top hub gene is CDK1, which indicates that the cell cycle will play a key role in TAD. The Cytoscape plugin MCODE results also showed that the first prognostic pathway was the cell cycle. The results of the two analysis methods are consistent and in accordance with previous studies [8–10].

Endothelial cells and SMCs are important in maintaining vascular tone in blood vessels. A previous study confirmed that restricting the cell cycle of endothelial cells and SMCs might lead to disordered vascular remodeling as an antecedent to the pathological sequelae of cardiovascular disease [31]. The SMC functional changes are major pathogenic factors of TAD. SMC progression and proliferation are critical for the development of TAD. Therefore, most studies aim to research the cell cycle of SMCs [32]. Proliferation arrest of SMCs in G0/G1 and G2/M-phases and inhibition of cyclin B1 expression and CDK1 activity (essential for G2-to-M progression) may protect against vascular-proliferative diseases. Other investigations also revealed an important role for VSMC proliferation in aortic tissue degeneration, which is believed to initiate pathologic remodeling in TAD [33]. Abnormal proliferation and migration of VSMCs and changes in the cell cycle have been proven to be the main cause of pathological vascular remodeling through undermining vasculature stability and finally leading to vascular disease. Many studies have aimed to research VSMCs from dissected aortas, and these SMCs can proliferate more rapidly than normal VSMC tissues. Therefore, the genes participating in proliferation exhibited increased expression. In our study, the 10 hub genes were all upregulated, showing that upregulated expression of these genes plays an important role in TAD, which is consistent with a previous study. In addition, we examined an mRNA profile (GSE116434) to determine if CDK1 also presented with high expression. However, the hub gene CDK1 was not significantly differentially expressed in a mouse model of TAD.

In summary, by means of high-throughput sequencing and data processing as well as qRT-PCR validation and immunohistochemistry staining, CDK1 may have potential as a drug target and diagnostic marker of TAD. The cell cycle may be the key pathway in TAD. However, there are still some limitations: use of normal aortic tissue as a control group may be more accurate. Further experimental studies with larger sample sizes are needed to confirm the role of the cell cycle pathway in TAD. Differential expression of the hub gene CDK1 between abdominal aortic aneurysm and TAD should also be verified.

## Methods

### Tissue collection

This study was conducted in accordance with the Declaration of Helsinki and was approved by the Ethics Committee of the Second Hospital of Jilin University. Ascending aortic specimens near the intimal tear were obtained from TAD patients undergoing surgical repair (n = 6) if informed consent was obtained. Aortic specimens derived from patients without aortic diseases undergoing coronary artery bypass (n = 6) graft were obtained as the no TAD (NT) group if informed consent was obtained. General information of all patients is shown in Table 3. There were no significant differences in age, gender, obesity, or smoking between the two groups. Patients diagnosed with TAD were confirmed by computed tomography angiography (CTA), and hereditary TAD was excluded. All specimens were immediately frozen in liquid nitrogen and preserved at -80°C for microarray analysis and qRT-PCR or further usage.

**Table 3:** General information for all patients.

	TAD group	NT group	P-value
	(n = 6)	(n = 6)	
Age (years old)	55.61±9.23	57.53±10.61	0.7450
Male	3 (50.00%)	3 (50.00%)	-
Obesity (BMI >25 kg/m2)	2 (33.33%)	3 (50.00%)	0.5582
Smoking	2 (33.33%)	4 (66.67%)	0.2482
NYHA class III-IV	1 (16.67%)	2 (33.33%)	0.5055
Hypertension	4 (66.67%)	3 (50.00%)	0.5582
Diabetes mellitus	1 (16.67%)	3 (50.00%)	0.2207
Chronic renal dysfunction	0	0	-

TAD: Thoracic aortic dissection; NT: no TAD; NYHA: New York Heart Association

### RNA extraction and quality control

Tissue RNA from the TAD and NT groups was extracted according to the manufacturer’s instructions by TRIzol reagent (Invitrogen, NY, USA). A NanoDrop ND-1000 (Thermo Fisher Scientific, Wilmington, DE, USA) and an Agilent 2100 Bioanalyzer (Agilent Technologies) were used to assess the integrity and concentrations of the RNA samples. All qualified samples were stored at -80°C for future experiments.

### Microarray analysis

Twelve samples (6 TAD and 6 NT) were used for microarray analysis by an Arraystar Human mRNA microarray, which contained 14596 mRNA probes. Sample preparation and array hybridization were performed according to the Agilent One-Color Microarray-Based Gene Expression Analysis protocol (Agilent). Briefly, mRNA was purified from total RNA (RNeasy Mini Kit, Qiagen) after removal of rRNA and transcribed into fluorescent cRNA without 3' bias along the entire length of the transcripts with a random priming method. A Quick-Amp Labeling Kit (Agilent, USA) was used for sample labeling. Next, a NanoDrop ND-1000 was used to determine the specific activity and concentration of the labeled cRNAs. Then, hybridization was performed in an Agilent Hybridization Oven. Data normalization and processing were performed using the GeneSpring GX v12.1 software package (Agilent). After quantile normalization of the raw data, mRNA samples with flags in the presence or margin (“All Targets Value”) were chosen for further data analysis. The differentially expressed mRNAs were statistically significant, with the change in threshold values >2.0 or < −2.0-fold between the two groups and Benjamini-Hochberg corrected P <0.05.

### GO and pathway enrichment analyses

The DAVID online bioinformatics database is an analysis tool for biological data to integrate and provide information for biological function and protein lists [34]. This tool was used in this study to provide GO enrichment and KEGG pathway analysis. GO analysis included the CC, BP, and MF categories. Pathway analysis is a functional analysis that maps genes to KEGG pathways. Gene counts >2 and p < 0.05 were set as the cutoff point.

### Integration of PPI network analysis

STRING (https://string-db.org/cgi/input.pl) is an online database resource search tool that can provide analysis of interacting genes, including physical and functional associations [35]. In this study, the STRING online tool was used to construct a PPI network of upregulated and downregulated DEGs, with a confidence score >0.7 defined as significant. Then, the interaction data were assessed using Cytoscape software [36] to structure a PPI network. Based on the above data, we used MCODE [37], a built-in APP in Cytoscape software, to analyze the interaction relationships of the DEGs encoding proteins and to screen hub genes. The parameters of network scoring and cluster finding were set as follows: degree cutoff = 2, node score cutoff = 0.2, k-core = 2, and max depth = 100.

### qRT-PCR

#### Validation and statistical analysis

qRT-PCR was used to verify the core genes. Total RNA was reverse-transcribed to cDNA using the PrimeScript RT Reagent Kit with gDNA Eraser (TaKaRa, Japan) according to the manufacturer’s instructions. Primer 5.0 software (PREMIER Biosoft, Palo Alto, CA, USA) was used to design primers, and a QuantStudio 7 Flex real-time PCR system (Applied Biosystems, Carlsbad, CA, USA) was used. All primers used in this study are listed in Table 4. All samples were normalized to GAPDH. The relative expression levels of each gene were calculated using the 2^-Δ Δ Ct^ method.

**Table 4:** The primers of the top 10 hub genes.

Gene name	Forward primer	Reverse primer
CDK1	TTGGCTGCTTTGAAAGTCTACG	GGTATGGTAGATCCGCGCTAAA
CDC20	ATGCGCCAGAGGGTTATCAG	AGGATGTCACCAGAGCTTGC
CCNB2	TCCAAAGGGTCCTTCTCCCA	TTGCAGAGCAAGGCATCAGA
CCNB1	GAAACGCATTCTCTGCGACC	AGCATTAATTTTCGAGTTCCTGGT
MAD2L1	CGTGCTGCGTCGTTACTTTT	GCCGAATGAGAAGAACTCGG
AURKA	GGATATCTCAGTGGCGGACG	GCAATGGAGTGAGACCCTCT
C3AR1	CAGTGAGGAGCTCACACGTT	TAAGAGCCCCTGCTTGTTGG
NCAPG	GCCCATTGTTACTGTTGGTGTT	TGCAATGTTTCAGCATCATTCTTCT
CXCL12	CTGTGCCCTTCAGATTGTAGCC	AAAGTGTGCAAAACAAAGCCCT
ASPM	AGAGTTAATGCAGCACTCGTCA	CCTCCACATAGCCTGAATAAGTGA
GAPDH	CGGACCAATACGACCAAATCCG	AGCCACATCGCTCAGACACC

### Immunohistochemistry staining

All samples were fixed in 4% neutral formaldehyde solution and embedded in paraffin. Tissue blocks were sliced into 2 μm sections and dewaxed, hydrated and antigen-repaired by PT link (Dako, Agilent Technologies, USA). Specifically, the slices were placed in repair solution preheated to 65°C and incubated for 30 minutes by heating to 90°C, then cooled to 70°C. Subsequently, the slices were washed by PBS. Incubation with primary and secondary antibodies and DAB coloring solution was automated by an Autostainer Link 48 (Dako, Agilent Technologies, USA). Specifically, the slices were incubated with hydrogen peroxide for 10 minutes, primary antibody for 30 minutes, and secondary antibody for 20 minutes at room temperature, followed by counterstaining with hematoxylin, routine dehydration, transparency, and sealing.

### Statistical analysis

The data are presented as the mean ± standard deviation. Statistical analyses were performed using SPSS version 20.0 (SPSS Inc., Chicago, IL, USA). The raw data were preprocessed using the affy package in R software and the limma package in R software. Comparisons between groups were performed using unpaired Student’s t-test. Fisher’s exact test was used to evaluate the significance of GO terms and Pathway identifier enrichment. False discovery rate (FDR) controlling was used to correct the P-values. FDR and P-values less than 0.05 were considered statistically significant.

## Abbreviations

TAD: thoracic aortic dissection
CAD: coronary heart disease
ECM: extracellular matrix
VSMC: vascular smooth muscle cell
GWAS: Genome-wide Association Studies
NT: no thoracic aortic dissection
CTA: computed tomography angiography
GO: Gene Ontology
KEGG: Kyoto Encyclopedia of Genes and Genomes
CC: cellular component
BP: biological processes
MF: molecular function
PPI: Protein-Protein Interaction
DEGs: differentially expressed genes
MCODE: Molecular Complex Detection
qRT-PCR: quantitative reverse transcription-PCR
FDR: false discovery rate
CDK: cyclin-dependent kinase
